# How Does Genetic Information Enable Life?

**DOI:** 10.1007/s11538-026-01636-0

**Published:** 2026-04-06

**Authors:** Robert Gatenby

**Affiliations:** Integrated Mathematical Oncology Department, Moffitt Cancer Center, 12902 Magnolia Dr, Tampa, FL 33512 USA

**Keywords:** Shannon information, Genetics, Evolution, Enzymes, Thermodynamics, Non-equilibrium, Fine graining, Coarse graining

## Abstract

Life requires genetic information to maintain a stable, highly ordered state far from thermodynamic equilibrium, but the general principles and specific mechanisms governing these dynamics are not well established. Here, the role of information in maintaining the unique thermodynamic state of living systems is examined in enzyme-accelerated reactions. Thermodynamically, reaction rates are governed by temperature and activation energy in the empirically derived Arrhenius equation. Living systems use genetically encoded enzymes to accelerate reactions up to 15 orders of magnitude without increased temperature. This is quantified in the Arrhenius equation as decreased activation energy but achieved physically by optimizing quantum interactions of substrate molecules to increase the probability of reaction. This scale transition from molecular mechanics in the information encoded amino acid sequence to quantum mechanics in substrate interactions represents “fine graining” which, as the opposite of coarse graining, requires added information. This is hypothesized to emerge from the cell’s molecular machinery that controls folding kinetics to ensure (with high probability) the genetically encoded string of amino acids folds to a single enzymatically functional 3-dimensional configuration from all other thermodynamically possible states thus increasing Shannon information. Enzyme-accelerated reactions alter concentrations of substrate and products without increased temperature to generate a Boltzmann distribution that is highly improbable for, and therefore, not in equilibrium with the cell’s thermodynamic state (temperature). Failure to maintain this non-equilibrium results in death, enabling evolutionary feedback. Furthermore, since protein function governs organism fitness, evolutionary selection is applied to both the gene that encodes the protein and cellular mechanisms that control its folding. By altering probabilistic quantum states during chemical reactions and producing statistical mechanics (Boltzmann distribution) inconsistent with the cellular thermodynamic state, the probability functions of Shannon information in the genome act at microscopic/macroscopic interfaces to enable the ordered, non-equilibrium state necessary for life.

## Introduction

Living systems are remarkably diverse but share a common thermodynamic state that is stable and highly ordered while far from equilibrium (Schrödinger 1944). Life uses inherited genetic information and acquired energy to maintain this non-equilibrium state and failure results in death with loss of its heritable information—dynamics that produce evolution (Vanchurin et al. 2022).

The explicit link of genetics and information theory was initially recognized by Fisher (1918) and Wright (1926). The non-random sequence of nucleotides in the DNA is typically quantified as Shannon information (Nemzer 2017), which measures the informational content of transmitted messages (Zeeberg 2002) but not the actual function or meaning of that information (Gatenby and Frieden 2013, 2007; Frieden and Gatenby 2011, 2021). Thus, information theoretic analysis can quantify the complexity of the genome but does not define the fundamental principles and specific mechanisms that allow genetic information to produce the low-entropy, non-equilibrium thermodynamic state (Gatenby and Frieden 2007) that is the unique property of life.

The challenge in translating “Shannon entropy” in the genome to cellular thermodynamic “entropy” is readily evident in “unit matching”. Because Shannon entropy is based on probability functions, it has no physical units (i.e., measured in bits) while thermodynamic entropy has units of temperature and energy (Joules per Kelvin (J/K)).

Here, the interface of genetic information and cellular thermodynamics is explored in the function of enzymes. At a macroscopic (thermodynamic) scale, intracellular reactions are described by the Arrhenius equation (see below) and governed by temperature and activation energy while, at the microscopic scale, the reaction is governed by probabilistic quantum interactions. In the absence of an enzyme, the rate of any given reaction can be increased only by injecting heat to raise the temperature. Genetically encoded information in an enzyme increases the reaction rate often by 10^5^−10^12^ s^−1^ within the narrow temperature range that is compatible with life. Heat is released by the reactions but usually dissipated into the environment without a significant temperature change. Within the Arrhenius equation, enzymatic acceleration of reactions is quantified as a decreased energy of activation. However, it is physically achieved by binding the reactants and altering their quantum interaction to increase the probability of a reaction (Sutcliffe and Scrutton 2000). Thus, the thermodynamic “value” of the genetic information in the enzyme can be estimated by the corresponding reduction of activation energy (Gatenby and Frieden 2016) but this provides no specific mechanism.

Here, the probabilistic Shannon information in enzymes is proposed to act upon statistical dynamics that govern microscopic states (quantum mechanics and Boltzmann distribution) to alter their corresponding observable macroscopic properties (reaction rates and entropy). These functions at the interface of microscopic/macroscopic scales integrate information (Barnett and Probability and Information 2020), thermodynamics (Eigen and Entropy and Information 2013), and evolution to enable life.

## Information Theory and Thermodynamics

Information theory emerged in the early twentieth century in part motivated by a thought experiment posed by Maxwell in the mid-nineteenth century (Maxwell 1871) when thermodynamic principles were developed by Clausius and others and corresponding microscopic states characterized by Boltzmann (Gao et al. 2019).

Briefly, Maxwell proposed a “finite being” (commonly described as “Maxwell’s demon”) could control a frictionless door between two chambers containing gas at the same temperature. The demon observes the velocity of gas molecules approaching the door and opens it to allow only fast-moving molecules to pass through. Because the temperature of the gas in each box is dependent on the Boltzmann distribution of velocities in the constituent molecules, the demon causes the temperature of one chamber to increase while the other decreases—an apparently “spontaneous” decrease in entropy. Maxwell viewed the demon *gedanken* as a violation of the second law of thermodynamics and subsequent analysis has generally focused on this conclusion.

In the early twentieth century, Szilard (1925) and Brillouin (1959), working separately, noted the demon’s function requires information, i.e., measurement of the gas molecule’s velocity. Thus, from its origin, information theory was deeply connected with thermodynamics. This was theoretically formalized by Landauer (1961) who quantified the energy dissipated as heat when information is deleted (an irreversible process). This cost has been experimentally confirmed by Berut et al. (2012) and Toyabe et al. (2010).

While Maxwell’s *gedanken* has elicited often contentious debates (Earman and Norton 1999), its application to biology has been limited. Haldane (1930) and Cohen and Monod (1957) first noted the similarity of enzyme and molecular receptor dynamics to Maxwell’s demons. As summarized by Mizraji, “biological Maxwell’s demons can be broadly viewed as information catalysts with thermodynamics consequences” (Mizraji 2021).

An important but generally unrecognized component of Maxwell’s thought experiment is that the demon acts at the interface of the microscopic state of a system, characterized by the Boltzmann distribution of molecular velocities, with its macroscopic (thermodynamic) state defined by temperature and entropy. That is, the demon uses information to alter the distribution of microstates in each chamber (increasing the average velocity in one and decreasing it in the other). When viewed as a thermodynamic system, it appears there has been spontaneous flow of heat from one chamber to the other—an apparent violation of thermodynamic principles. However, Maxwell’s assertion reflects a traditional top-down approach in which a change in the thermodynamic state (i.e. by changing temperature) alters the microstates of the components of the system (i.e., Boltzmann distribution of velocities). In contrast, Maxwell’s demon acts in a bottom-up manner by using information to alter the statistical distribution of micro-states in each chamber and thereby producing a change in its observable, macroscopic (thermodynamic) state.

Thus, Maxwell’s *gedanken* is, at its core, an exploration of the function of information at the macroscopic-microscopic interface of systems that can be described by both classic thermodynamics (e.g. entropy, heat, and energy) and statistical physics (Teschendorff and Feinberg 2021). As outlined below, life fundamentally uses genetic information to exploit this interface.

*Biological Information*

Heritable information in the genome of living systems is a linear sequence of discrete nucleotide triplets that encodes a corresponding sequence of amino acids in proteins that then form the structural and functional components of a cell. Because the functions of the proteins are determined by the specific order of the amino acids, the information content of the corresponding non-random sequence of nucleotide triplets can be quantified using the discrete form of Shannon information (Akhter et al. 2013):

1 $$ H\left( X \right) = - \mathop \sum \limits_{x \in X} p\left( d \right)\log p\left( x \right) $$

where *H(X)* represents Shannon “entropy” (17–19), *X* is a set of discrete variables (*x*_*1*_*, x*_*2*_*,,,,,x*_*n*_) that represent all possible states of the system (for example, the sequence of nucleotide triplets in a gene) each defined by probability *p(x*) which, therefore, represents the fraction of the whole population in this state. Shannon information (*S*_*I*_) increases as the content of a message (here the genome) deviates from randomness.

2 $$ S_{I} = - k_{B } \sum p_{i} \ln p_{i} $$

where *S*_*I*_ is Shannon or information entropy, *k*_*B*_ is the Boltzmann constant, and *p*_*i*_ is the probability of each microstate. Shannon used the term “entropy” because of its formal similarity to thermodynamic entropy in which a system that has *i* possible microstates, thermodynamic entropy *(S*_*T*_) is

3 $$ S_{T} = - k_{B} \ln \Omega $$

where *Ω* is the number of microstates that can produce the observed macroscopic system so that a change in entropy over time is:

4 $$ \Delta S_{T} = k_{B} \ln \left( {\frac{{\Omega_{final} }}{{\Omega_{initial} }}} \right) $$

Thus, over time, changes in microstates within the system are collectively expressed as a change in entropy.

Shannon information is often framed as a measure of “surprise”. That is, improbable states that are, therefore, more surprising contain greater information than those states that are more probable and less surprising (Schneider 2006; Palm 2012). This reflects a fundamental link between Shannon information and randomness and statistics. A state that is entirely random is statistically most probable and has no Shannon information. More improbable states of the system contain more information and thus require information to be generated from a random state.

The Shannon (information) entropy of individual proteins is estimated to be about 2.5 bits per amino acid (Strait and Dewey 1996) and the human genome contains about 30 billion bits of Shannon information (Chang et al. 2005). While these calculations indicate a high level of order, they cannot be directly translated into the specific protein function or the thermodynamic state of an organism.

## The Microscopic/Macroscopic Interface: Coarse and Fine Graining

As noted above, the units of information (bits) are based on probability functions and do not directly correspond to units in thermodynamic equations, nor do they appear in the dynamics of chemical reactions characterized by the Arrhenius equation. However, both the Arrhenius equation and thermodynamics represent a macroscopic, coarse grained approximation of microscopic dynamics that can be characterized statistically. That is, in chemical reactions, the Arrhenius equation represents a coarse graining of a sequence of transitional quantum states characterized by a reaction path and probability functions governed by the Doob-Gillespie algorithm (Gillespie 1977). Similarly, thermodynamic entropy is the macroscopic and coarse grained manifestation of microstates characterized by statistical mechanics, (e.g., Boltzmann distributions) (Enders 2021).

## Information at the Molecular/Quantum Interface in Enzymes

Life accelerates critical reaction rates using genetically encoded enzymes:

$$ {\text{S }} + {\text{ E}} \to {\text{ ES }} \to {\text{ E }} + {\text{ P}} $$

where S is substrate; E, enzyme; ES, the enzyme- substrate complex; P, the reaction product.

**Fig. 1:**
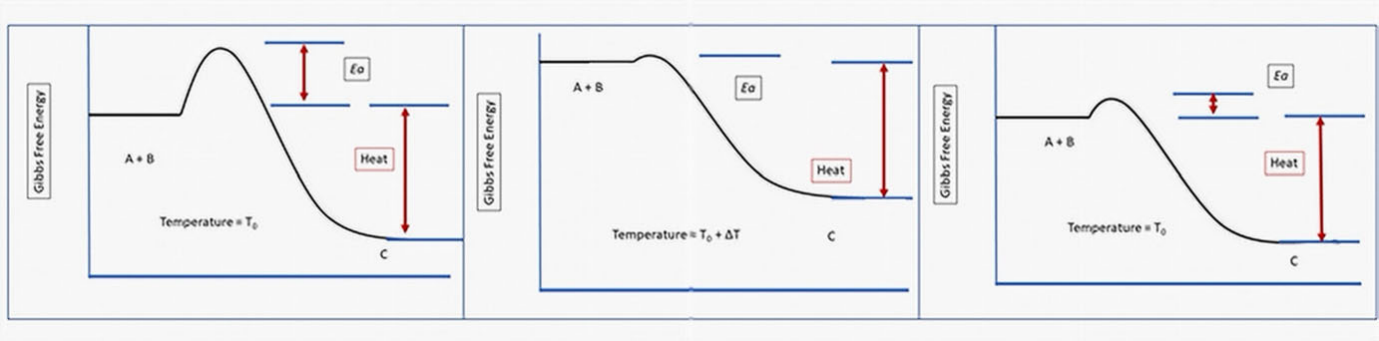
The dynamics of accelerating the chemical reaction A + B → C through injection of heat. The y axis is the Gibbs free energy, and the x axis is “reaction coordinates”. Left panel shows conventional diagram showing the reaction can spontaneously occur because the Gibbs free energy of the product is lower than that of the reactants. The reaction rate is governed by the activation energy, which is a fixed constant that is independent of the thermodynamic state of the system. Middle panel, the reaction is accelerated by the influx of heat which raises the energy of both the reactants and product. Because the activation energy is not changed by the increased temperature, the reaction is accelerated. However, the energy states of the reactants and products are higher. Right panel, addition of an enzyme has the effect of reducing the activation energy but, since an influx of heat is not required, the reaction is accelerated without any change in energy levels of the reactants or product (note, the heat produced by the reaction is typically assumed to dissipate into the environment and neglected in the models). Thus, the final state of both systems that accelerate a reaction may have the same concentration of reactants and products, its thermodynamic state is quite different

In non-living systems, the reaction rate is dependent on the temperature and activation energy (Fig. 1) as defined by the empirically derived Arrhenius equation:

5 $$ k = Ae^{{\frac{ - Ea}{{RT}}}} $$

where *K* is the reaction rate, *A* is a constant, *E*_*a*_ is activation energy, *R* is the universal gas constant and *T* is temperature in Kelvin. The heat production from the reaction is typically assumed to be dissipated into the environment and neglected in this model.

In the thermodynamic model of reactions, injecting heat into the system will increase the temperature (T) and, therefore, the reaction rate However, living systems are typically constrained to narrow temperature ranges limiting their ability to accelerate reaction rates by influx of heat. Instead, living systems employ heritable (genetic) information in the form of enzymes that accelerate chemical reactions up to 15 orders of magnitude (Cooper 2019). This effect is expressed thermodynamically as a reduction of the activation energy in the Arrhenius equation (Gatenby and Frieden 2016).

However, the specific mechanism of enzymatic acceleration is optimization of the quantum interactions of substrate molecules to increase the probability of a reaction (Sutcliffe and Scrutton 2000; Dutton et al. 2006; Naray-Szabo et al. 2013). Enzymes function by capturing the reactants and placing them in close proximity (Alberts 2015) and precise orientation to resemble a reaction transition state (Cooper 2019). This minimum separation of their quantum wave functions (von Neumann entropy (Molina-Espiritu et al. 2015)) was described by Pauling as an “enhanced transition state (Pauling 1948)” and may include quantum tunneling (Brookes 2017). In 2013, the Chemistry Nobel Prize was awarded to Warshel, Levitt, and Karplus for developing theoretical and computational methods to investigate the quantum mechanisms in the active site of an enzyme (Foundation 2013). Here, computational models distinguish quantum mechanics (QM) at the enzyme’s active site from Molecular Mechanics (MM) that govern the interactions at other sites in the protein (Mehmood and Kulik 2022; Karelina and Kulik 2017).

The transition from molecular mechanics (MM) in the amino acid constituents of the protein to quantum mechanics (QM) at its active site represents a macroscopic → microscopic transition broadly defined as “fine graining” (Sinitskiy and Voth 2018). Briefly, coarse and fine graining reflect variations in the granularity of a system. Coarse graining, represented by thermodynamics and the Arrhenius equation, smooths the fine grained structures so that, for example, multiple microstates (e.g. a progression of quantum states in reaction or the Boltzmann distribution of velocities of gas molecules in a gas at some temperature) can be characterized by a single large scale (macro) state (e.g. temperature or reaction rate). Coarse graining is commonly used to simplify complex systems but loss of fine structures results in decreased information and increased entropy (Maroney 2009). Because fine graining investigates system microstates that are not observable on the macroscopic scale, it requires addition of information (Maroney 2009).

An entertaining analogy by Susskind (2013), is that of a cotton ball. The ball itself is a coarse-grained manifestation of the complex interactions of the cotton fibers within the ball. “Coarse graining” simplifies the description of complex systems generally by describing them in larger spatial or temporal scales. This loss of fine structure is thermodynamically favorable because it increases entropy but with a cost of decreased information (Shell 2016). On the other hand, moving from larger to smaller spatial scales (“fine graining “) decreases entropy and can be achieved only through addition of information. In the cotton ball analogy, for example, fine graining requires information about the distribution of fibers that give rise to but are not observable in its macroscopic (spherical, white, etc.) appearance.

How is this added information obtained?

## Protein Folding and Information Gain in Living Systems

The genetically encoded one-dimensional amino acid string can function as an enzyme only by assuming 3-dimensional configuration that allows it to bind substrate molecules and alter their quantum interactions. Protein folding is thermodynamically favored because it lowers the free energy. However, there are typically multiple potential low free energy final structures for the encoded enzymes, which, on average, consist of about 200 amino acids (see below) but can exceed 2000 (Robinson 2015).

**Fig. 2:**
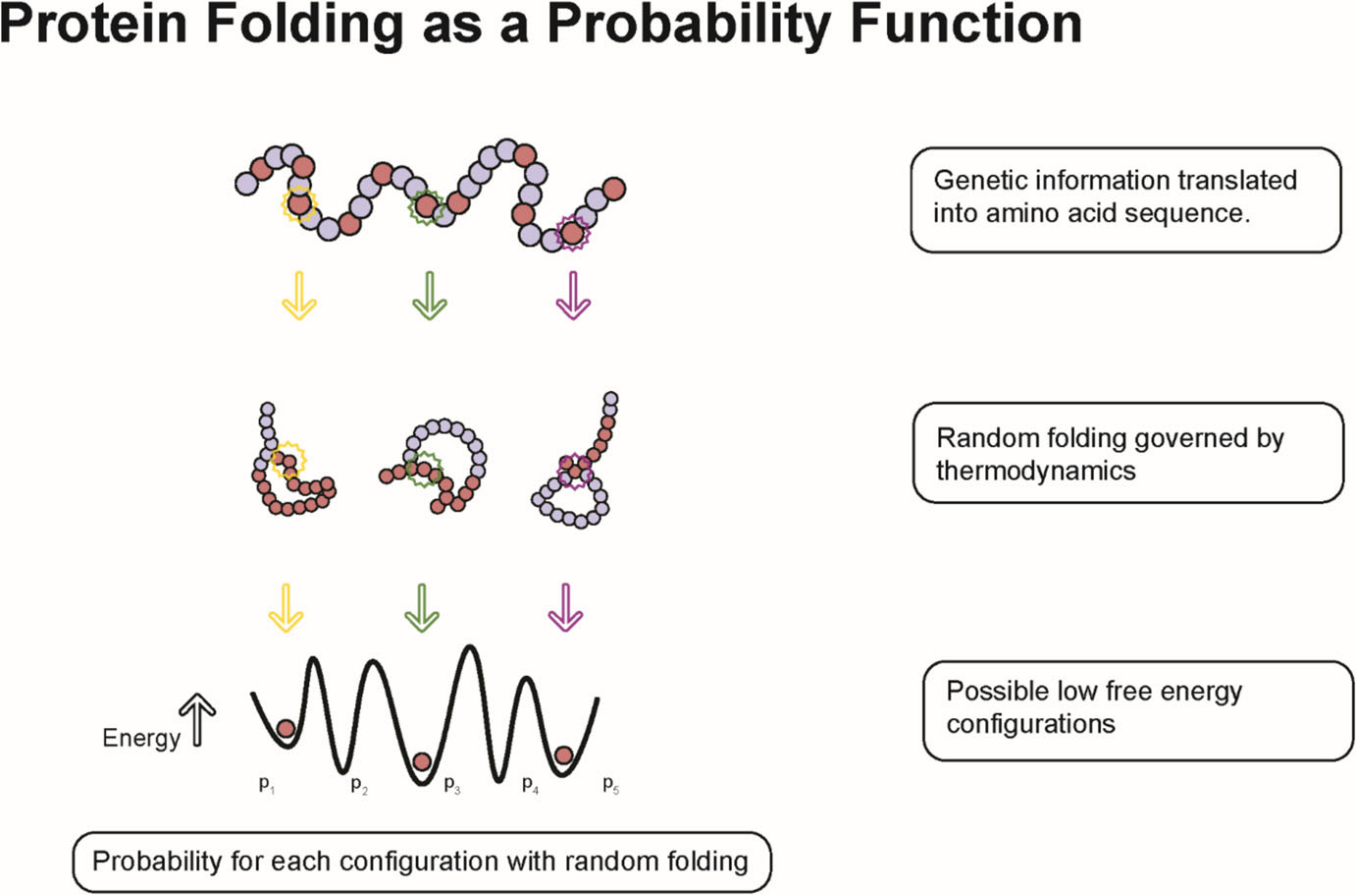
The encoded genetic information is translated into a string of amino acids. However, biological function of this information requires folding of protein into a specific active 3-dimensional configuration. The folding process to a lower free energy state results in decreases the entropy of the polypeptide with release of heat. Governed solely by the local thermodynamic interactions, the complex folding process can result in multiple final configurations—commonly viewed as a free energy funnel but presented here as a 2-dimensional state. Depending on the thermodynamic state, all the possible 3-dimensional states can be characterized by some probability. Living systems, by controlling the kinetics of protein folding, can consistently (but not entirely error-free) produce a specific, maximally functional final state. As demonstrated in the text, this improbably outcome represents a gain in information

The process of protein folding is complex often with multiple transition steps resulting in a final 3-dimensional structure determined by interactions among the constituent amino acids and subject to variations in local condition and stochastic events (Dill et al. 2008). If each polypeptide had only one possible 3-dimensional state, protein folding would not alter the cellular information state. However, for most amino acid sequences there are multiple potential states with a corresponding probability distribution (Fig. 2) that may vary with cytoplasmic conditions including temperature, pH, and ion concentrations.

Levinthal (1968) first noted that a protein of 100 peptides has up to 3^200^ possible configurations and that protein folding in cells proceed far more quickly (microseconds to minutes) than expected (termed “Levinthal’s paradox”). Thus, protein folding governed solely by a free energy landscape is often viewed as a funnel leading to multiple free energy minimum states that can result in multiple configurations, each of which has some probability of occurring depending on local conditions (Dobson 2003) (Fig. 2).

However, living systems control the kinetics of folding (Baker and Agard 1994) to enforce (but with some errors—“misfolded proteins”) a single functional 3 dimensional configuration. Mechanisms of control include variations ribosome structure (Streit et al. 2024; Kaiser et al. 2011; Cassaignau et al. 2020) (the human genome encodes 80 different ribosomal proteins (Uechi et al. 2001)) and diverse chaperone proteins (Saibil 2013; Hartl et al. 2011)) to generate a specific 3-dimensional configuration that performs the necessary function (Balchin et al. 2016) even when thermodynamically unfavorable (Baker and Agard 1994; Sorokina et al. 2022). Here, the free energy difference between the final protein state and the more favorable thermodynamic state, based on the Jarzynski equality (Jarzynski 1997), represents the work applied by the protein folding machinery. Specific examples of these interactions include protein entanglement in which short chains from different proteins can interact to alter the folding probability (see folding of RD1 protein and role of SH3 domain see Fig. 1 in Salicari et al. (2023)) and functions of the “exit tunnel” in ribosomes(Javed et al. 2017).

Thus, transition from a 1-dimensional string of amino acids to a specific folded, functional 3-dimensional protein out of many possible thermodynamic states represents an improbable outcome and, therefore, gain of Shannon information (*I*). That is, if protein folding under any given thermodynamic conditions can result in *n* possible configurations (*c*_*1*_*, c*_*2*_*, c*_*3*_* … c*_*n*_) each of which has some probability *p(c*_*1*_*), p(c*_*2*_*), p(c*_*3*_*) … p(c*_*n*_*).* The probability distribution allows protein folding to be framed in the context of Shannon information:

6 $$ I_{C} \left( c \right) = {\mathrm{log}}\left( {\frac{1}{{P_{C} \left( c \right)}} } \right) $$

where *C* represents all possible 3-dimensional protein configurations for any given amino acid sequence with each configuration *c* defined by a probability *p(c)*. Living systems, by controlling the folding kinetics, ensure that, under normal conditions, a single, optimally functioning enzyme configuration (with rare misfolding errors) is reached out of all possible states. By Eq. 6, this highly improbable outcome represents an increase in Shannon information within the framework of Jarzynski equality (Jarzynski 1997; Wilson and Correa 2025):

7 $$ I_{C} \left( c \right) = {\mathrm{log}}\left( {p\left( {c_{notf} } \right)} \right) - {\mathrm{log}}\left( {p\left( {c_{f} } \right)} \right) $$

where ${c}_{f}$ is the maximally functional 3-dimensional configuration that is enforced by kinetic control of folding and ${c}_{notf}$ are all other thermodynamically possible configurations. Here, *p* is the probability these configurations will result from protein folding governed only by thermodynamic mechanisms.

The gain of information during protein folding can be interpreted as the result of communication between amino acids during the folding process. That is, the encoded information is maintained as a rigid line by both DNA and mRNA so that there is no communication between elements of the encoded message. Here, the information dynamics may be analogous to written words. That is, the distribution of letters in a word or words in a Shakespear play can be used to calculate its Shannon information. However, the meaning of the word or the themes of the play emerge from interactions among the letter and words, respectively. In this word/sentence analogy, transition from one to three dimensions during protein folding that permits communication among amino acids is equivalent to permitting interactions of letters within a word and words within a sentence permitting hermeneutic circles (Martin 1972) to form global meaning.

The thermodynamic “balance sheet” is maintained because the increase in information corresponds to heat released during protein folding (Galano-Frutos and Sancho 2024), which can range (depending on the protein size) from 60 to 900 kcal/mol (Seelig and Seelig 2022).

This gain of information is hypothesized to be necessary for the fine graining that allows the molecular dynamics that govern enzyme structure to act upon the quantum interactions of substrate at its active site. Of note, enzyme proteins are generally large (Srere 1984). Assuming there is a generally direct correlation between the number of amino acids and number of protein configurations, more information can be obtained in larger proteins (see below) but probably at an added cellular cost for an increase in the necessary molecular machinery needed to maintain control of larger polypeptides.

These dynamics also add a layer of complexity to evolutionary selection. That is, Darwinian dynamics act upon the interactions of the heritable phenotype with environmental selection forces and, thus, the *function* of the encoded enzymes. As a result, evolutionary selection is applied both to the gene and to the broader cellular properties related to folding of its encoded protein. The latter adds a layer of complexity in which the evolutionary benefit of optimal protein folding is balanced against cost of the molecular machinery of folding within the energy availability to the cell.

## Information at the Statistical Physics/Thermodynamic Interface in Living Systems

How does genetic information, manifested as an increased reaction rate, produce the non-equilibrium cellular state necessary for life?

**Fig. 3:**
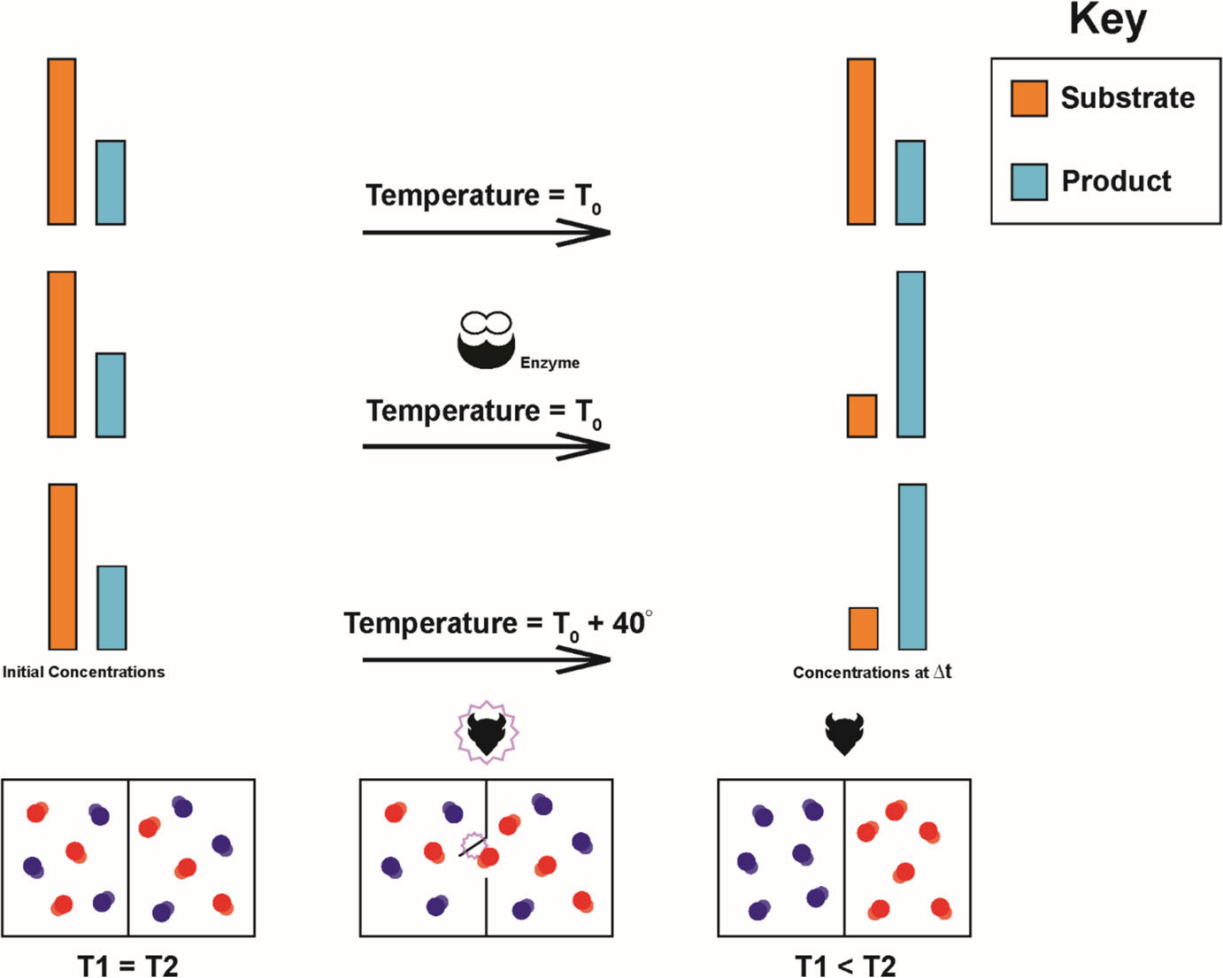
Lower Panels demonstrate the macroscopic/microscopic interface of a system in which the macroscopic state is measured by temperature and the corresponding macroscopic state is characterized by a Boltzmann distribution of velocities in constituent molecules. Injection of heat into the system increases the temperature and produces a Boltzmann distribution with higher velocities. Enzymes can accelerate biological reactions up to 15 orders of magnitude. In the absence of enzymes, chemical reactions are accelerated by an increased temperature. Upper panels demonstrate reactions can be accelerated either by the function of an enzyme which alters the quantum interactions of substrate molecules or through injection of heat to increase temperature. Enzymes, by rapidly altering the Boltzmann distribution of substrate and products, produces a Boltzmann distribution equal to but in the absence of a temperature increase. This “bottom up” change produces a thermodynamic non-equilibrium state. Lower Panels demonstrate the actions of an enzyme mimic the “bottom up” approach of the demon in Maxwell’s famous “*demon gedanken*”. That is, by altering segregating fast-moving molecular in one box and slow-moving molecules in the other, the demon alters the Boltzmann distribution. Maxwell noted that this would produce a decreased entropy and an apparent flow of heat between isothermal components of the system and, therefore, an apparent violation of thermodynamic laws. In this context, demon simply alters the macroscopic property of the system by using information and energy to change its microscopic state – entirely consistent with thermodynamics principles

As noted above, genetic information in enzymes accelerates multiple intracellular reactions by as much as 15 orders of magnitude. About 4.2 × 10^6^ proteins/cell are found in *Saccharomyces cerevisiae* of which 20–40% are enzymes (Ho and Brown 2018). The collective function of this large number of enzymes has global effect of consuming reactants and increasing the concentration of products thus altering Boltzmann and local microstates (Jinwoo and Tanaka 2015) with a corresponding decreased entropy by Eq. 4. Thus, information acts upon cellular order/entropy through a “bottom up” (Jin et al. 2022) dynamics similar to Maxwell’s demon as described above (Fig. 3).

 By altering the local concentrations of substrate and products identical to but in the absence of increased temperature, the collective actions of intracellular enzymes generate a non-equilibrium state. That is, similar to Maxwell’s demon, biological information in enzymes alters the Boltzmann distribution of intracellular molecules to one that is highly improbable state (i.e., with low concentrations of substrate and high concentration or products) for its thermodynamic environment (i.e., local temperature) (Fang et al. 2019). To be clear, Maxwell’s demon (as noted above) is used to illustrate the interaction of information at the microscopic/macroscopic interface of a complex system and does not imply a violation of thermodynamic principles. That is, information generates increased order by exporting entropy into the environment—dynamics that are entirely consistent with thermodynamic laws in an open system.

Living systems are required to continuously use information to maintain this non-equilibrium state. Failure to do so results in death enabling evolutionary selection.

## Discussion

Life uses heritable information and acquires energy to maintain a stable, ordered state that is far from thermodynamic equilibrium. While the genome contains high levels of Shannon Information based on a statistically improbable distribution of nucleotides, this information has no physical units to directly allow a thermodynamic function. Analysis of these dynamics demonstrates that, as a general principle, statistically-derived biological information acts on microscopic components of a system that can be characterized statistically – quantum interactions governing chemical reactions and Boltzmann distributions in thermodynamic systems. By altering the quantum interactions of substrate molecules, genetic information in an enzyme increases the probability of a reaction thus accelerating the reaction rate equivalent to but in the absence of increased temperature. In turn, the accelerated reaction rate produces a Boltzmann distribution of reactants and products that are highly improbable for the thermodynamic state (i.e. temperature) of the cell thus producing non-equilibrium conditions.

An added complexity emerges because genetic information is molecularly encoded in a sequence of nucleotides but must act upon quantum level dynamics to accelerate a reaction. This fine graining requires additional information which can be produced when the polypeptide folds into a 3-dimensional shape. When folding is governed solely by thermodynamical interactions, a large number of possible 3-dimensional (including many non-functional) configurations will be produced, each of which can be characterized by some probability (Fig. 2). However, by controlling the kinetics of protein folding, living systems enforce a single final configuration that is optimally functional—a statistically improbable outcome that, therefore, represents a gain of Shannon information at the thermodynamic cost of heat released during folding (Galano-Frutos and Sancho 2024). This increased information allows the fine graining necessary for molecularly encoded genetic information to act upon substrate quantum interactions in its active site.

**Fig. 4:**
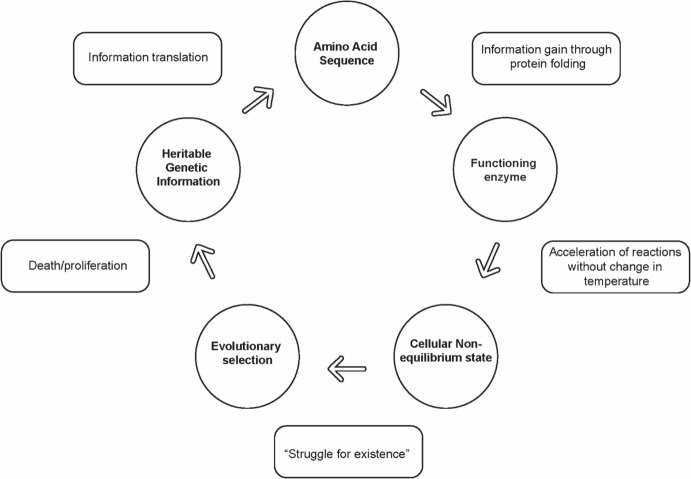
A summary of the information/thermodynamic/evolutionary dynamics at the microscopic/macroscopic interface. Genetic sequences permit storage of information that can be transmitted across generations, providing the Darwinian mechanism of inheritance. Molecularly encoded information in enzymes acts at a quantum level to accelerate reaction. This fine graining (macroscopic → microscopic transition) requires additional information obtained by control of protein folding. By accelerating reactions, the information in enzymes produces an intracellular Boltzmann distribution of reactant and product equivalent to but in the absence of increased temperature—a non-equilibrium state. A living system far from equilibrium must continuously employ information and energy to maintain its state or die thus generating the necessary Darwinian “struggle for existence”. Evolutionary selection governing proliferation or death then provides the feedback to promote optimal information dynamics including both the genetically encoded amino acid sequence and the intracellular dynamics that control protein folding

Similarly, the function of enzymes acts at the interface of statistical physics (Boltzmann distribution) and thermodynamics. By accelerating reactions, cellular enzymes collectively generate concentrations of substrate and products highly improbable for the environmental thermodynamic state thus generating non-equilibrium conditions. These bottom-up dynamics are analogous to Maxwell’s demon *gedanken* in which the demon produces a Boltzmann distribution that alters the entropy and temperature in components of the system. This does not violate thermodynamic laws (as hypothesized by Maxwell) but does produce a non-equilibrium state that can be maintained only by ongoing activity by the demon (i.e., continuous use of information plus the necessary energy, Fig. 3). Failure to maintain this non-equilibrium state results in death providing evolutionary feedback—a macroscopic → microscopic dynamic that “closes the loop” at the microscopic/macroscopic interface (Fig. 4).

These dynamics have potential implications for evolutionary principles. Cells must maintain extensive molecular machinery (e.g. ribosomes and chaperone proteins) that control the kinetics of protein folding to ensure a specific functional configuration (Dhar et al. 2011). Since evolutionary fitness of an organism is governed by the functions of its constituent proteins, Darwinian selection must apply to both the inherited gene and the broader cellular properties that control protein folding. This generates a cost/benefit trade-off in which the fitness benefits generated by the molecular machinery of protein folding are limited by the cost of encoding, expressing, and maintaining the necessary genes within the energy budget of the organism.

Kocher and Dill (2024) have pointed out protein folding is a critical dynamic in the origin of life, which requires genetic information to improve survival and proliferation of proto-life forms in fluctuating environmental conditions (Gatenby et al. 2025). A prediction of this model is that evolutionary selection, by increasing the reliability of protein folding would permit the size of proteins to generally increase over the history of life. This is, in fact, observed as, as average protein size in eukaryotes (472 aa) is great than bacteria (320 aa) and archaea (283 aa) (Tiessen et al. 2012). Greater lengths eukaryotic proteins are found in all functional categories and the nearly all protein families (Brocchieri and Karlin 2005). Similarly, protein number and size were significantly greater in multicellular compared to single cellular organisms (Tiessen et al. 2012).

Finally, the information gain of protein folding from 1 to 3 dimensions demonstrates a critical role for dimensionality in the link between genetic information and biological function and complexity. This may represent a broad principle in biology that is applicable, for example, to the development of a multicellular organism. Interestingly, while the human genome is large, containing about 30 billion bits (Chang et al. 2005), an adult human body contains 36 trillion cells (Hatton et al. 2023). Thus, the informational content of the genome must be significantly expanded to form a large, complex, multi-organ, multi-tissue functioning organism that is robust to a broad range of perturbations. Here, the fertilized egg is viewed as point source of information. As the fertilized egg proliferates to form a 3-dimensional multicellular tissue, information is gained through non-random interactions among the cells analogous to the interactions of amino acids during protein folding. Thus, the human body (and all stages of development to adulthood) represents a highly improbable configuration compared to all potentially 3-dimensional shapes if proliferation was simply a random process. This permits the information gain necessary for development of large, complex multicellular organisms and contrasts to the disordered tissue that results from cancer cell proliferation.

This theoretical model, while speculative, is supported by the identical multicellular tissue observed in *Choanoeca flexa* (Ros-Rocher et al. 2026) generated by coalescence of an existing cell population or proliferation of one cell within a population. That is, both produce a highly non-random multicellular structure with, therefore, increased Shannon and both are achieved through exchange of genetically encoded information among a pre-existing population of cells or a population that result from proliferation of a single progenitor cell. The proposed, genetically encode set of interactions, which is the biological mechanism for the dimensional expansion of information, is observed in genetic information patterns such as eigengenes (Kerstjens et al. 2026)—co-expression patterns of genes in adjacent cells—allow multi-scale information dynamics during development of complex tissue such as the brain (Wolpert 2016).

## Data Availability

All data generated or analyzed during this study are included in this published article.

## References

[CR1] Akhter S, Bailey BA, Salamon P et al (2013) Applying Shannon’s information theory to bacterial and phage genomes and metagenomes. Sci Rep 3:1033. 10.1038/srep01033 23301154 10.1038/srep01033 PMC3539204

[CR2] Alberts B (2015) Molecular biology of the cell. Garland Science, Taylor and Francis Group, New York, NY

[CR3] Baker D, Agard DA (1994) Kinetics versus thermodynamics in protein folding. Biochemistry 33(24):7505–7509. 10.1021/bi00190a002 8011615 10.1021/bi00190a002

[CR4] Balchin D, Hayer-Hartl M, Hartl FU (2016) In vivo aspects of protein folding and quality control. Science 353(6294):aac4354. 10.1126/science.aac4354 27365453 10.1126/science.aac4354

[CR5] Barnett S, Probability and Information (2020) Quantum Information. Oxford Academic, London

[CR6] Berut A, Arakelyan A, Petrosyan A et al (2012) Experimental verification of Landauer’s principle linking information and thermodynamics. Nature 483(7388):187–189. 10.1038/nature10872 22398556 10.1038/nature10872

[CR7] Brillouin L (1959) Information theory and its applications to fundamental problems in physics. Nature 183:501–502

[CR8] Brocchieri L, Karlin S (2005) Protein length in eukaryotic and prokaryotic proteomes. Nucleic Acids Res 33(10):3390–3400. 10.1093/nar/gki615 15951512 10.1093/nar/gki615 PMC1150220

[CR9] Brookes JC (2017) Quantum effects in biology: golden rule in enzymes, olfaction, photosynthesis and magnetodetection. Proceed Royal Soc: Math Phys Eng Sci 473(2201):20160822. 10.1098/rspa.2016.0822 10.1098/rspa.2016.0822 PMC5454345 28588400

[CR10] Cassaignau AME, Cabrita LD, Christodoulou J (2020) How does the ribosome fold the proteome? Annu Rev Biochem 89:389–415. 10.1146/annurev-biochem-062917-012226 32569518 10.1146/annurev-biochem-062917-012226

[CR11] Chang C-HHL-C, Chen T-Y, Chen H-D, Luo L, Lee H-C (2005) Shannon information in complete genomes. J Bioinf Comput Biol 3(3):1–22 10.1142/s0219720005001181 16108085

[CR12] Cohen GN, Monod J (1957) Bacterial permeases. Bacteriol Rev 21(3):169–194. 10.1128/br.21.3.169-194.1957 13471457 10.1128/br.21.3.169-194.1957 PMC180897

[CR13] Cooper GM (2019) The cell : a molecular approach. Sinauer Associates, an imprint of Oxford University Press, Oxford ; New York

[CR14] Dhar A, Girdhar K, Singh D et al (2011) Protein stability and folding kinetics in the nucleus and endoplasmic reticulum of eucaryotic cells. Biophys J 101(2):421–430. 10.1016/j.bpj.2011.05.071 21767495 10.1016/j.bpj.2011.05.071 PMC3136782

[CR15] Dill KA, Ozkan SB, Shell MS et al (2008) The protein folding problem. Annu Rev Biophys 37:289–316. 10.1146/annurev.biophys.37.092707.153558 18573083 10.1146/annurev.biophys.37.092707.153558 PMC2443096

[CR16] Dobson CM (2003) Protein folding and misfolding. Nature 426(6968):884–890. 10.1038/nature02261 14685248 10.1038/nature02261

[CR17] Dutton PL, Munro AW, Scrutton NS, Sutcliffe MJ (2006) Introduction Quantum catalysis in enzymes: beyond the transition state theory paradigm. Philos Trans R Soc Lond B Biol Sci 361(472):1293–1294. 10.1098/rstb.2006.1879

[CR18] Earman J, Norton JD (1999) Exorcist XIV: The wrath of Maxwell’s demon. Part II. From Szilard to Landauer and beyond. Stud Hist Philos Sci Part b: Stud Hist Philos Mod Phys 30(1):1–40. 10.1016/S1355-2198(98)00026-4

[CR19] Eigen M, Entropy and Information (2013) From strange simplicity to complex familiarity, a treatise on Matter, Information, Life and Thought. Oxford Academic, Oxford

[CR20] Enders P (2021) Statistical mechanics and thermodynamics: Boltzmann’s versus Planck’s state definitions and counting (dagger). Entropy (Basel) 23(7):875. 10.3390/e23070875 34356416 10.3390/e23070875 PMC8304142

[CR21] Fang X, Kruse K, Lu T, Wang J (2019) Nonequilibrium physics in biology. Rev Mod Phys 91:045004. 10.1103/RevModPhys.91.045004

[CR22] Fisher RA (1918) The correlation between relatives on the supposition of Mendelian inheritance. Trans R Soc Edinb 52:399–433

[CR23] Foundation TN The Nobel Prize in Chemistry. Accessed 2013

[CR24] Frieden BR, Gatenby RA (2021) Principle of minimum loss of fisher information, arising from the cramer-rao inequality; its role in evolution of bio-physical laws, complex systems and universes. In: Srinivasa A, RCR, Plastino AS (ed)*. *Information Geometry, Vol 45. Elsevier, p 420

[CR25] Frieden BR, Gatenby RA (2011) Information dynamics in living systems: prokaryotes, eukaryotes, and cancer. PLoS ONE 6(7):e22085. 10.1371/journal.pone.0022085 21818295 10.1371/journal.pone.0022085 PMC3139603

[CR26] Galano-Frutos JJ, Sancho J (2024) Energy, water, and protein folding: a molecular dynamics-based quantitative inventory of molecular interactions and forces that make proteins stable. Protein Sci 33(2):e4905. 10.1002/pro.4905 38284492 10.1002/pro.4905 PMC10804899

[CR27] Gao X, Gallicchio E, Roitberg AE (2019) The generalized Boltzmann distribution is the only distribution in which the Gibbs-Shannon entropy equals the thermodynamic entropy. J Chem Phys 151(3):034113. 10.1063/1.5111333 31325924 10.1063/1.5111333

[CR28] Gatenby RA, Frieden BR (2007) Information theory in living systems, methods, applications, and challenges. Bull Math Biol 69(2):635–657. 10.1007/s11538-006-9141-5 17083004 10.1007/s11538-006-9141-5

[CR29] Gatenby RA, Frieden BR (2013) The critical roles of information and nonequilibrium thermodynamics in evolution of living systems. Bull Math Biol 75(4):589–601. 10.1007/s11538-013-9821-x 23456476 10.1007/s11538-013-9821-x PMC4073208

[CR30] Gatenby R, Frieden BR (2016) Investigating information dynamics in living systems through the structure and function of enzymes. PLoS ONE 11(5):e0154867. 10.1371/journal.pone.0154867 27149068 10.1371/journal.pone.0154867 PMC4857929

[CR31] Gatenby RA, Gallaher J, Subramanian H et al (2025) On the origin of information dynamics in early life. Life. 10.3390/life15020234 10.3390/life15020234 PMC11856217 40003644

[CR32] Gillespie D (1977) Exact stochastic simulation of coupled chemical reactions. J Phys Chem 81(25):2340–2361. 10.1021/j100540a008

[CR33] Haldane JBS (1930) Enzymes. Longmans, Green, London, New York

[CR34] Hartl FU, Bracher A, Hayer-Hartl M (2011) Molecular chaperones in protein folding and proteostasis. Nature 475(7356):324–332. 10.1038/nature10317 21776078 10.1038/nature10317

[CR35] Hatton IA, Galbraith ED, Merleau NSC et al (2023) The human cell count and size distribution. Proc Natl Acad Sci U S A 120(39):e2303077120. 10.1073/pnas.2303077120 37722043 10.1073/pnas.2303077120 PMC10523466

[CR36] Ho BBA, Brown GW (2018) Unification of protein abundance datasets yields a quantitative *Saccharomyces cerevisiae* proteome. Cell Syst 6:1–14. 10.1016/j.cels.2017.12.004 29361465 10.1016/j.cels.2017.12.004

[CR37] Jarzynski C (1997) Nonequilibrium equality for free energy differences. Phys Rev Lett 78:2690–2693. 10.1103/PhysRevLett.78.2690

[CR38] Javed A, Christodoulou J, Cabrita LD et al (2017) The ribosome and its role in protein folding: looking through a magnifying glass. Acta Crystallogr D Struct Biol 73(Pt 6):509–521. 10.1107/S2059798317007446 28580913 10.1107/S2059798317007446 PMC5458493

[CR39] Jin J, Pak AJ, Durumeric AEP et al (2022) Bottom-up coarse-graining: principles and perspectives. J Chem Theory Comput 18(10):5759–5791. 10.1021/acs.jctc.2c00643 36070494 10.1021/acs.jctc.2c00643 PMC9558379

[CR40] Jinwoo L, Tanaka H (2015) Local non-equilibrium thermodynamics. Sci Rep 5:7832. 10.1038/srep07832 25592077 10.1038/srep07832 PMC4296294

[CR41] Kaiser CM, Goldman DH, Chodera JD et al (2011) The ribosome modulates nascent protein folding. Science 334(6063):1723–1727. 10.1126/science.1209740 22194581 10.1126/science.1209740 PMC4172366

[CR42] Karelina M, Kulik HJ (2017) Systematic quantum mechanical region determination in QM/MM simulation. J Chem Theory Comput 13(2):563–576. 10.1021/acs.jctc.6b01049 28068092 10.1021/acs.jctc.6b01049

[CR43] Kerstjens S, Engert F, Douglas RJ et al (2026) A lineage-based model of scalable positional information in vertebrate brain development. Neuron. 10.1016/j.neuron.2025.12.043 10.1016/j.neuron.2025.12.043 41775258

[CR44] Kocher CD, Dill KA (2024) Origins of life: the protein folding problem all over again? Proc Natl Acad Sci U S A 121(34):e2315000121. 10.1073/pnas.2315000121 39133848 10.1073/pnas.2315000121 PMC11348307

[CR45] Maldacena J, Susskind L (2013) Cool horizons for entangled black holes. 10.48550/arXiv.1306.0533

[CR46] Landauer R (1961) Irreversibility and heat generation in the computing process. IBM J Res Dev 5(3):183–191. 10.1147/rd.53.0183

[CR47] Levinthal C (1968) Are there pathways for protein folding? J Med Phys 65:44–45

[CR48] Maroney E (2009) Information processing and thermodynamic entropy. In: Zalta ENs (ed) The Stanford Encyclopedia of Philosophy. Stanford, CA, Metaphysics Research Lab, Stanford University

[CR49] Martin W (1972) The hermeneutic circle and the art of interpretation. Comp Lit 24(2):97–117

[CR50] Maxwell JC (1871) Theory of Heat. Longmans, Green, and Co, London, UK

[CR51] Mehmood R, Kulik HJ (2022) Quantum-mechanical/molecular-mechanical (QM/MM) simulations for understanding enzyme dynamics. Methods Mol Biol 2397:227–248. 10.1007/978-1-0716-1826-4_12 34813067 10.1007/978-1-0716-1826-4_12

[CR52] Mizraji E (2021) The biological Maxwell’s demons: exploring ideas about the information processing in biological systems. Theory Biosci 140(3):307–318. 10.1007/s12064-021-00354-6 34449033 10.1007/s12064-021-00354-6 PMC8568868

[CR53] Molina-Espiritu MER, Lopez-Rosa S, Deshesa JS (2015) Quantum entanglement and chemical reactivity. J Chem Theory Comput. 10.1021/acs.jctc.5b00390 10.1021/acs.jctc.5b00390 26894237

[CR54] Naray-Szabo G, Olah J, Kramos B (2013) Quantum mechanical modeling: a tool for the understanding of enzyme reactions. Biomolecules 3(3):662–702. 10.3390/biom3030662 24970187 10.3390/biom3030662 PMC4030948

[CR55] Nemzer LR (2017) Shannon information entropy in the canonical genetic code. J Theor Biol 415:158–170. 10.1016/j.jtbi.2016.12.010 28007553 10.1016/j.jtbi.2016.12.010

[CR56] Palm G (2012) Novelty, information and surprise. Springer, Heidelberg; New York

[CR57] Pauling L (1948) Chemical achievement and hope for the future. Am Sci 36:51–58 18920436

[CR58] Robinson PK (2015) Enzymes: principles and biotechnological applications. Essays Biochem 59:1–41. 10.1042/bse0590001 26504249 10.1042/bse0590001 PMC4692135

[CR59] Ros-Rocher N, Reyes-Rivera J, Horo U et al (2026) Clonal-aggregative multicellularity tuned by salinity in a choanoflagellate. Nature. 10.1038/s41586-026-10137-y 10.1038/s41586-026-10137-y PMC13017551 41741645

[CR60] Saibil H (2013) Chaperone machines for protein folding, unfolding and disaggregation. Nat Rev Mol Cell Biol 14(10):630–642. 10.1038/nrm3658 24026055 10.1038/nrm3658 PMC4340576

[CR61] Salicari L, Baiesi M, Orlandini E et al (2023) Folding kinetics of an entangled protein. PLoS Comput Biol 19(11):e1011107. 10.1371/journal.pcbi.1011107 37956216 10.1371/journal.pcbi.1011107 PMC10681328

[CR62] Schneider TD (2006) Claude Shannon: biologist. The founder of information theory used biology to formulate the channel capacity. IEEE Eng Med Biol Mag 25(1):30–33. 10.1109/memb.2006.1578661 10.1109/memb.2006.1578661 PMC1538977 16485389

[CR63] Schrödinger E (1944) What is life? The physical aspect of the living cell. Cambridge University Press, Cambridge, England

[CR64] Seelig J, Seelig A (2022) Molecular understanding of calorimetric protein unfolding experiments. Biophys Rep (n y) 2(1):100037. 10.1016/j.bpr.2021.100037 36425081 10.1016/j.bpr.2021.100037 PMC9680786

[CR65] Shell MS (2016) Coarse-graining with the relative entropy. In: Rice SA, DARs (ed) Advances in chemical physics, John Wiley and Sons, Inc.

[CR66] Sinitskiy AV, Voth GA (2018) Quantum mechanics/coarse-grained molecular mechanics (QM/CG-MM). J Chem Phys 148(1):014102. 10.1063/1.5006810 29306280 10.1063/1.5006810

[CR67] Sorokina I, Mushegian AR, Koonin EV (2022) Is protein folding a thermodynamically unfavorable, active, energy-dependent process? Int J Mol Sci. 10.3390/ijms23010521 10.3390/ijms23010521 PMC8745595 35008947

[CR68] Srere PA (1984) Wye are enzymes so big? Trends Biochem Sci 9(9):387–390. 10.1016/0968-0004(84)90221-4

[CR69] Strait BJ, Dewey TG (1996) The Shannon information entropy of protein sequences. Biophys J 71(1):148–155. 10.1016/S0006-3495(96)79210-X 8804598 10.1016/S0006-3495(96)79210-X PMC1233466

[CR70] Streit JO, Bukvin IV, Chan SHS et al (2024) The ribosome lowers the entropic penalty of protein folding. Nature 633(8028):232–239. 10.1038/s41586-024-07784-4 39112704 10.1038/s41586-024-07784-4 PMC11374706

[CR71] Sutcliffe MJ, Scrutton NS (2000) Enzymology takes a quantum leap forward. Philos Trans R Soc Lond A Math Phys Eng Sci 358(1766):367–386. 10.1098/rsta.2000.0536 10.1098/rsta.2000.0536 PMC2854803 20396604

[CR72] Szilard L (1925) Über die Ausdehnung der phänomenologischen Thermodynamik auf die Schwankungserscheinungen. Z Phys 32(1):753–788. 10.1007/BF01331713

[CR73] Teschendorff AE, Feinberg AP (2021) Statistical mechanics meets single-cell biology. Nat Rev Genet 22(7):459–476. 10.1038/s41576-021-00341-z 33875884 10.1038/s41576-021-00341-z PMC10152720

[CR74] Tiessen A, Perez-Rodriguez P, Delaye-Arredondo LJ (2012) Mathematical modeling and comparison of protein size distribution in different plant, animal, fungal and microbial species reveals a negative correlation between protein size and protein number, thus providing insight into the evolution of proteomes. BMC Res Notes 5:85. 10.1186/1756-0500-5-85 22296664 10.1186/1756-0500-5-85 PMC3296660

[CR75] Toyabe SST, Ueda M, Sano M (2010) Experimental demonstration of information-to-energy conversion and validation of the generalized Jarzynski equality. Nat Phys 6:988–992

[CR76] Uechi T, Tanaka T, Kenmochi N (2001) A complete map of the human ribosomal protein genes: assignment of 80 genes to the cytogenetic map and implications for human disorders. Genomics 72(3):223–230. 10.1006/geno.2000.6470 11401437 10.1006/geno.2000.6470

[CR77] Vanchurin V, Wolf YI, Koonin EV et al (2022) Thermodynamics of evolution and the origin of life. Proc Natl Acad Sci USA 119(6):e2120042119. 10.1073/pnas.2120042119 35131858 10.1073/pnas.2120042119 PMC8833196

[CR78] Wilson CAM, Correa CG (2025) On the free energy of protein folding in optical tweezers experiments. Biophys Rev 17(2):231–245. 10.1007/s12551-025-01310-0 40376413 10.1007/s12551-025-01310-0 PMC12075763

[CR79] Wolpert L (2016) Positional information and pattern formation. Curr Top Dev Biol 117:597–608. 10.1016/bs.ctdb.2015.11.008 26970003 10.1016/bs.ctdb.2015.11.008

[CR80] Wright S (1926) A frequency curve adapted to variation in percentage occurrence. J Am Stat Assoc 21:162–178

[CR81] Zeeberg B (2002) Shannon information theoretic computation of synonymous codon usage biases in coding regions of human and mouse genomes. Genome Res 12(6):944–955. 10.1101/gr.213402 12045147 10.1101/gr.213402 PMC1383734

